# Genetic alterations in juvenile cervical clear cell adenocarcinoma unrelated to human papillomavirus

**DOI:** 10.3389/fmed.2023.1211888

**Published:** 2023-08-16

**Authors:** Yuehui Su, Yiming Zhang, Mengjiao Zhou, Ruijin Zhang, Siang Chen, Lili Zhang, Hao Wang, Dongdong Zhang, Ting Zhang, Xinqiang Li, Chunyan Zhang, Bingjie Wang, Shuyu Yuan, Mengzhuo Zhang, Yingying Zhou, Lili Cao, Mengzhen Zhang, Jianjun Luo

**Affiliations:** ^1^Department of Gynecology, First Affiliated Hospital of Zhengzhou University, Zhengzhou, China; ^2^Key Laboratory of RNA Biology, Institute of Biophysics, Chinese Academy of Sciences, Beijing, China; ^3^College of Life Sciences, University of Chinese Academy of Sciences, Beijing, China; ^4^Key Laboratory of Epigenetic Regulation and Intervention, Chinese Academy of Sciences, Beijing, China

**Keywords:** genetic alterations, cervical clear cell adenocarcinoma, human papillomavirus, CMTM5, juveniles

## Abstract

Clear cell adenocarcinoma of the cervix (CCAC) is a special type of HPV-independent cervical cancer. It has a low incidence rate, can be difficult to diagnose early, has a poor prognosis. Its peak incidence is in adolescence, which poses a great threat to women’s health. Therefore, it is very important to explore the pathogenesis of cervical clear cell adenocarcinoma to guide subsequent treatment and prevention. This study analyzed 3 juvenile patients with CCAC diagnosed at the First Affiliated Hospital of Zhengzhou University. Using next-generation sequencing methods, we analyzed the pathogenesis of the patients and their close relatives by analyzing the genetic alterations of patients. CMTM5 was identified as the only shared mutated gene. Using published literature and comparative analyses of related disease-causing genes, 6 of the 19 genes (ALKBH7, MYCBP, MZF1, RNF207, RRS1, and TUSC2) were screened as genes with mutations in patients and had higher mutation rates in reproductive cancers. Pathway analysis showed that downregulated genes in non-HPV cervical cancer were mainly related to the immune system response, suggesting that non-HPV cervical cancer differs from HPV-infected cervical cancer in that the immune response is weaker, which is consistent with the weak correlation with viral infection.

## Introduction

Cervical cancer is one of the most common gynecological malignancies. The latest statistics show that in developing countries, the morbidity and mortality of cervical cancer are second only to breast cancer (1). In recent years, the incidence of cervical cancer in China has been increasing, ranking first among female reproductive system tumors (2, 3). The vast majority of cervical cancers are caused by persistent infection with high-risk human papillomavirus (HPV), known as HPV-associated cervical cancer. HPV-associated cervical cancer mortality has gradually declined with the global increase in HPV vaccination coverage and the development of HPV testing as a primary screening technique, allowing many early-stage lesions to be screened and treated. However, according to the fifth edition of the World Health Organization’s (WHO) classification of female reproductive tumors, nearly 10% of cervical cancers are not related to HPV infection, mainly some rare pathological types (4), called HPV-independent cervical cancers (5, 6). Cervical cancer that does not rely on HPV is easily missed and misdiagnosed, and it is often detected at an advanced stage, which affects treatment and prognosis.

Clear cell adenocarcinoma of the cervix (CCAC) is a rare and highly characteristic cervical adenocarcinoma with a low incidence, accounting for only 3% of all cervical adenocarcinomas (7). In recent years, the incidence rate has been increasing, particularly in younger patients (8). The pathogenesis of CCAC is still unclear; it is prone to early metastasis and is associated with a worse prognosis than other types of cervical cancer, which seriously affects the physical and mental health of women. Here, we retrospectively analyzed the clinical data and whole genome sequencing data of three cases of juvenile HPV-independent CCAC and comprehensively analyzed and summarized their clinical characteristics, genetic alterations, related etiology, and pathogenesis.

## Materials and methods

### Patient information

Case 1: patient-1, female, negative HPV test, no family history of cervical cancer, no sexual history or diethylstilbestrol exposure *in utero*, was admitted to the hospital at the age of 14 because of “irregular vaginal bleeding for more than 1 year” (Supplementary Table S1). Hysteroscopy + tumor resection was performed, and clear cell carcinoma was considered in postoperative pathology. The chemotherapy regimen was nedaplatin + Lipusu. The patient underwent two chemotherapy treatments in our hospital, and the chemotherapy effect was good. Later, she was transferred to a higher-level hospital for surgery, radiotherapy and chemotherapy. After 6 months, due to poor treatment effects, the disease progressed, and the patient died.

Case 2: patient-2, female, negative HPV test, no family history of cervical cancer, no sexual history or diethylstilbestrol exposure *in utero* (Supplementary Table S1). At the age of 6 years, she was hospitalized due to “abnormal vaginal discharge for one and a half years and a small amount of vaginal bleeding for more than 1 month.” Postoperatively, a PT regimen (docetaxel + oxaliplatin) was given for four courses of chemotherapy, and no obvious abnormality was found in regular follow-up. Three years later, she was readmitted to the hospital due to “vaginal bleeding.” Combined with the results of imaging examinations (with photos) and biochemical examinations, the patient underwent “laparoscopic hysterectomy + bilateral adnexectomy + pelvic lymph node dissection.” The postoperative pathology showed CCAC, and six rounds of chemotherapy and a PT program (docetaxel + lobaplatin) were given after surgery. A 6 years follow up, the patient is still alive and regular re-examination has not indicated any signs of recurrence.

Case 3: patient-3, female, negative HPV test, no family history of cervical cancer, no sexual history or diethylstilbestrol exposure *in utero* (Supplementary Table S1). At the age of 14, she was admitted to our hospital due to “1 month of vaginal bleeding.” Due to the large lesion area, chemotherapy was performed with docetaxel + lobaplatin. After three rounds of chemotherapy, the lesion shrank significantly, and color Doppler ultrasound showed that the lesion range was approximately 25 × 12 mm. “Laparoscopic wide hysterectomy + bilateral adnexectomy + pelvic lymph node dissection” was performed. The postoperative pathology showed cervical clear cell carcinoma, and the diagnosis was cervical clear cell carcinoma. After a 4 years follow-up, the patient is still alive and regular re-examination has not indicated any signs of recurrence.

### Bioinformatics data analysis

In total, data from 149 cervical squamous cell carcinoma and endocervical adenocarcinoma (CESC) patients and 18,161 RNAs extracted from RNA-seq data were analyzed for transcriptome profiling. All RNA expression data were obtained from TCGA (August 30, 2022). The R packages DESeq2 (9) and WGCNA (10) were used to analyze differential genes and gene correlation with non-HPV infection.

FastQC^1^ was used for quality control of sequences. Whole genome sequencing reads were aligned to the human genome (hg38) with the aligner BWA-MEM (11). We then performed local realignment of the aligned reads with those high-quality InDels using the Genome Analysis Toolkit (GATK). GATK HaplotypeCaller and IndelRealigner were used to call SNPs, indels, and variants. The R package VCFR was used to read variant call format data in R, and circlize was used to visualize the results (12).

GO (Gene Ontology) enrichment analyses and visualization as well as KEGG (Kyoto Encyclopedia of Genes and Genomes) enrichment analyses and visualization of identified genes were performed by the clusterProfiler (13) R package with *p* < 0.05 as the cutoff value. A volcano plot was visualized by the ggplot2 package (version 3.3.5). Gene expression and disease-free survival analysis in TCGA CESC datasets were performed by GEPIA (14).

## Results

### Pathological features of juvenile CCAC independent of HPV

**Figure 1:**
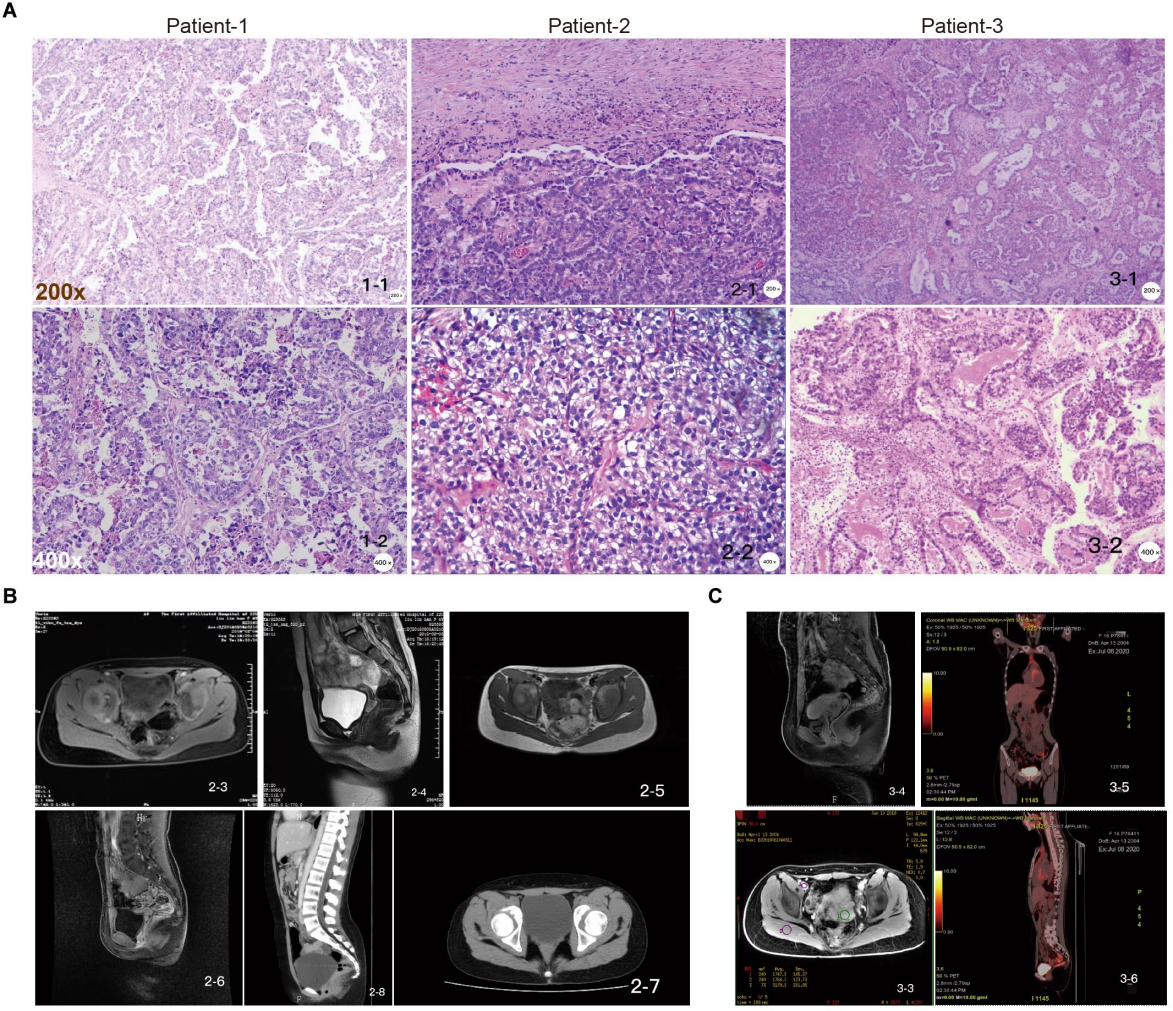
Pathologic examination of juvenile CCAC independent of HPV. **(A)** Patient-1, papillary growth areas with relatively small and regular papillae, sometimes larger papillae, with fibrovascular axis, tumor composed of moderately to severely atypical cells with clear cytoplasm and polygonal cells; patient-2, the tumor was composed of moderately to severely atypical cells with clear cytoplasm and polygonal cells; patient-3, in the papillary growth area, the papilla are small and relatively regular, sometimes larger, with fibrovascular axis, “shoe spike” cells, and cells protruding into the lumen of the gland. Scale: top row: 200×, bottom row: 400×. **(B)** Pelvic MRI images of patient-2 at initial diagnostic stages (2-3, 2-4); suggestive of recurrence stages (2-5, 2-6); and follow-up CT images showing no signs of recurrence (2-7, 2-8). **(C)** Pelvic MRI images of patient-3 at initial diagnosis (3-3, 3-4); follow-up PET-CT images showed no signs of recurrence (3-5, 3-6).

The common clinical manifestations of CCAC include irregular vaginal bleeding and fluid discharge. Patients with more advanced disease may experience abdominal pain, fever, pelvic mass and other uncomfortable symptoms. Tumors are usually located within the cervical canal and may have an exophytic or endophytic appearance. Over the past 5 years, we have treated three cases of juvenile cervical cancer, all with a negative HPV test, no family history of cervical cancer, and no sexual history or diethylstilbestrol exposure *in utero*. Histologically, they appear as relatively homogeneous tubular, papillary, or solid structures with cuboidal, flattened, or nail-like morphology, hyaline or eosinophilic cytoplasm, and nuclei with prominent high-grade features, such as hyperchromasia, polymorphism and prominent nucleoli (Figure 1A).

Gynecological examination of patient-1 showed a palpable irregular mass in the pelvis, with medium quality and moderate activity, approximately 4 × 3 cm in size (Figure 1A). MRI of the pelvis identified malignant lesions, suggesting vaginal or cervical cancer involving the vagina and was later diagnosed as a cervical mass (data not shown). The pathological consultation for patient-1, combined with the immunohistochemical results, was consistent with CCAC (Figure 1A). For patient-2, gynecological examination showed a palpable nodule approximately 2 × 3 cm in size on the right side of the upper vagina. The surface was soft and uneven, and the left ligament was thickened. Pelvic ultrasonography showed a hypoechoic upper vaginal segment close to the internal os of the cervix. Pelvic MRI showed possible cervical cancer, and biopsy showed clear cell carcinoma of the cervix. The postoperative pathology was consistent with cervical clear cell carcinoma, and the diagnosis was cervical clear cell carcinoma stage IIB (Figures 1A,B). For patient-3, gynecological examination revealed a hard mass in the upper part of the vagina, approximately 6 cm in size, inactive, without thickening of the parametrium. A CT scan before admission showed a mass (62 × 42 mm) in the cervix and upper vagina, which may be clear cell carcinoma after pathological consultation in our hospital. Some cells had well-defined borders and were “shoe spikes” (Figures 1A,C). The results of blood biochemical examination of 3 patients showed that markers such as SCCA, CA125, CA19-9, AFP, CEA, and HE-4 were all within the normal range.

### Genome sequencing and analyses revealed exceptional structural variation and genetic alterations

**Figure 2:**
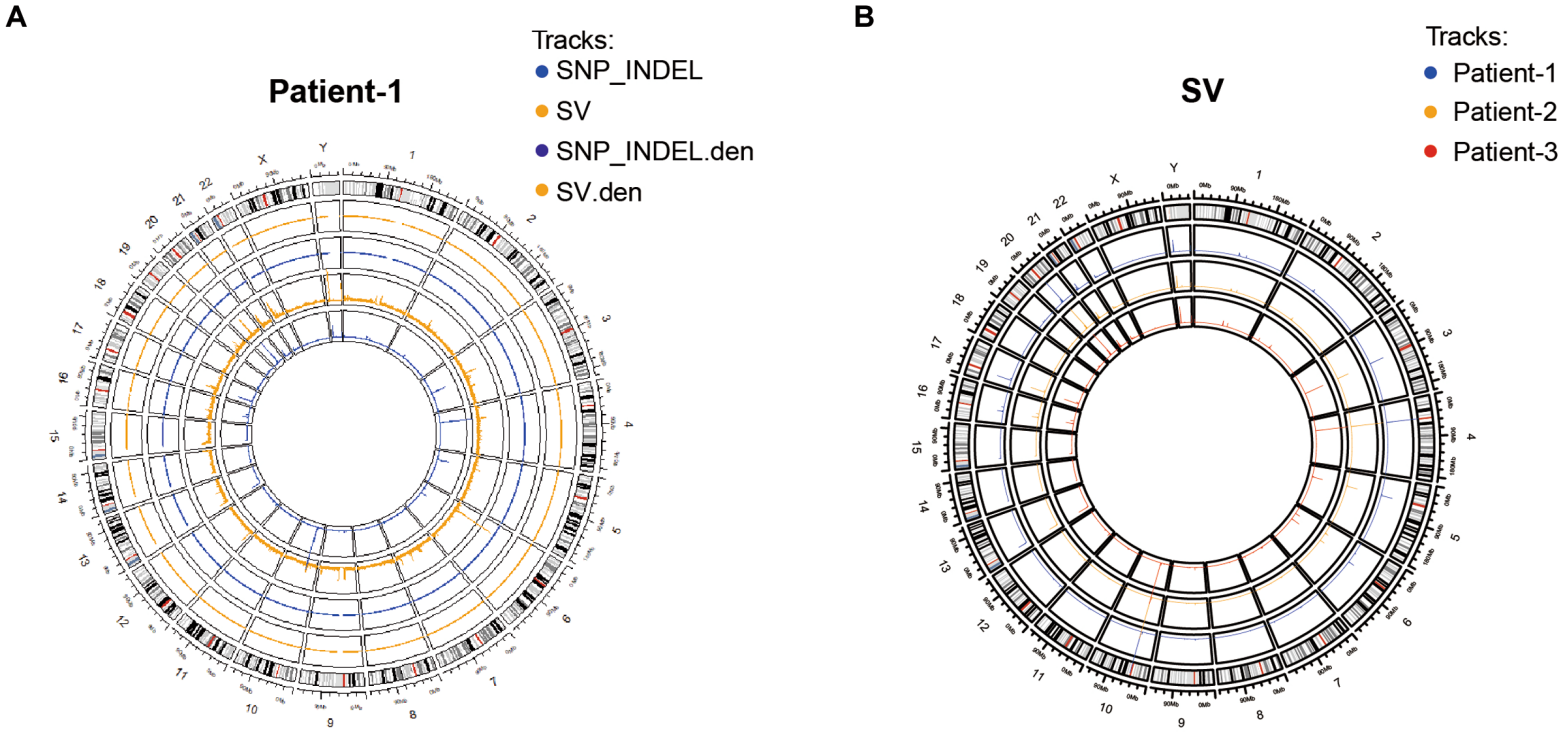
Characterization of genetic variants in three adolescent patients. **(A)** Distribution of SNP/INDEL and SV variants of patient-1. **(B)** Distribution of SV variation in three patients.

To systematically examine the genetic alterations underlying HPV-independent cervical cancer inheritance, whole genome sequencing was performed on peripheral blood mononuclear cell (PBMC) samples from these three adolescent patients and their parents. By analyzing the structural variation and gene mutation of these samples (Figure 2), 118 SNP or indel mutation genes different from their parents were detected in adolescent patients, including 86 genes of patient-1, 19 genes of patient-2, and 14 genes of patient-3 (Supplementary Table S1). Thus, these results generated an atlas of genetic alterations that will be valuable for further investigation of adolescent HPV-independent cervical cancer.

### Comparative analysis of transcriptome in non-HPV-infected cervical cancer

**Figure 3:**
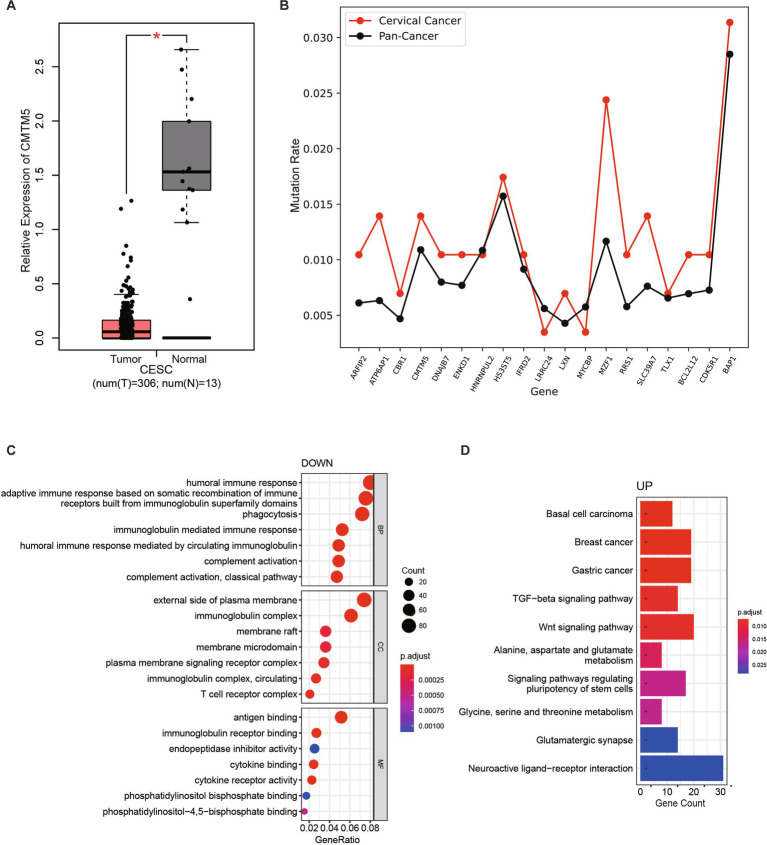
Comparative transcriptome analysis of non-HPV-infected cervical cancer. **(A)** Data from TCGA and GTEx were analyzed to compare CMTM5 expression levels in cervical cancer and normal samples, the gene expression levels were shown as log_2_(TPM+1). ^*^p < 0.05. **(B)** The mutation rates (mutated samples/total number of samples) of selected genes were compared between cervical cancer samples and all cancers in the TCGA database. **(C)** GO enrichment analysis of the most differentially downregulated pathways. **(D)** KEGG pathways enriched for the most differentially upregulated pathways.

From the generated genetic variation profile, we found that CKLF-like MARVEL transmembrane domain-containing member 5 (CMTM5) was the only gene mutated simultaneously in patient-1 and patient-3 (Supplementary Tables S2, S3). CMTM is a new protein family connecting chemokines and the transmembrane-4 superfamily (TM4SF), which plays an important role in the immune system, male reproductive system and tumorigenesis (15). In humans, they are encoded by nine genes, CKLF and CMTM1-8. CMTM5, located at 14q11.2, is a polygenic locus associated with a variety of tumors (16, 17). Frequent loss of heterozygosity at 14q11.2 in nasopharyngeal carcinoma (NPC) suggests the presence of a functional tumor suppressor gene (TSG) in this region. Another study confirmed that ~40% of downregulated genes were located at 1p and 14q in anaplastic meningiomas, whereas the 14q11.2 gene NDRG2 was consistently downregulated in grade III meningiomas (17). CMTM5 has at least six RNA spliced forms (CMTM5-v1-v6). In tumor cell lines, the CMTM5 promoter is frequently methylated, which can be reversed by pharmacological demethylation. Colony formation experiments showed that CMTM5-v1 repair in PC-3 and HeLa cells strongly inhibited cell growth. CMTM5-v1 inhibits cell proliferation and migration by downregulating the EGFR signaling pathway in prostate cancer. Considering the chromosomal location and protein structure of CMTM5, CMTM5 tends to be involved in tumorigenesis. Compared with normal samples, the expression of CMTM5 in cervical cancer samples was significantly downregulated, suggesting that CMTM5 plays an important role in the occurrence and development of cervical cancer (Figure 3A; Supplementary Figure S1A).

In addition, the mutation rate of some of the identified mutated genes in patients in The Cancer Genome Atlas (TCGA) database was further compared with the mutation rate of all cancers (mutated samples/total number of samples). Most of the specific mutation genes in these three patients had a higher mutation rate in cervical cancer compared with the average mutation rate in all tumors, indicating that mutations in these genes may be more likely to cause cervical cancer (Figure 3B). To further assess whether mutated genes affect cervical carcinogenesis, we incorporated the cervical squamous cell carcinoma (CESC) transcriptome dataset from TCGA. In total, this study analyzed data from 149 CESC patients with available expression data and clinical information in the TCGA database (data version, August 30, 2022). Comparative analysis of differentially expressed genes (DEGs) between 143 HPV-infected samples and six non-HPV-infected samples revealed 1,748 upregulated genes and 1,861 downregulated genes (Supplementary Figure S1B). Using the “clusterProfiler” R package, 1,551 GO terms and 60 Kyoto Encyclopedia of Genes and Genomes (KEGG) terms were identified. The results based on the top seven downregulated GO terms of biological processes, cellular components, and molecular functions (Figure 3C), seven upregulated GO terms (Supplementary Figure S1C), and the top 10 upregulated KEGG terms (Figure 3D) showed that DEGs were mainly associated with the immune response, cytokine binding, TGF-beta and the Wnt signaling pathway. Genes downregulated in non-HPV cervical cancers were primarily related to the immune system response, suggesting that non-HPV cervical cancers differ from HPV-infected cervical cancers by a weaker immune response, consistent with the weak correlation with viral infection.

### Comprehensive analysis of important genes and their functions in non-HPV-associated cervical cancer

**Figure 4:**
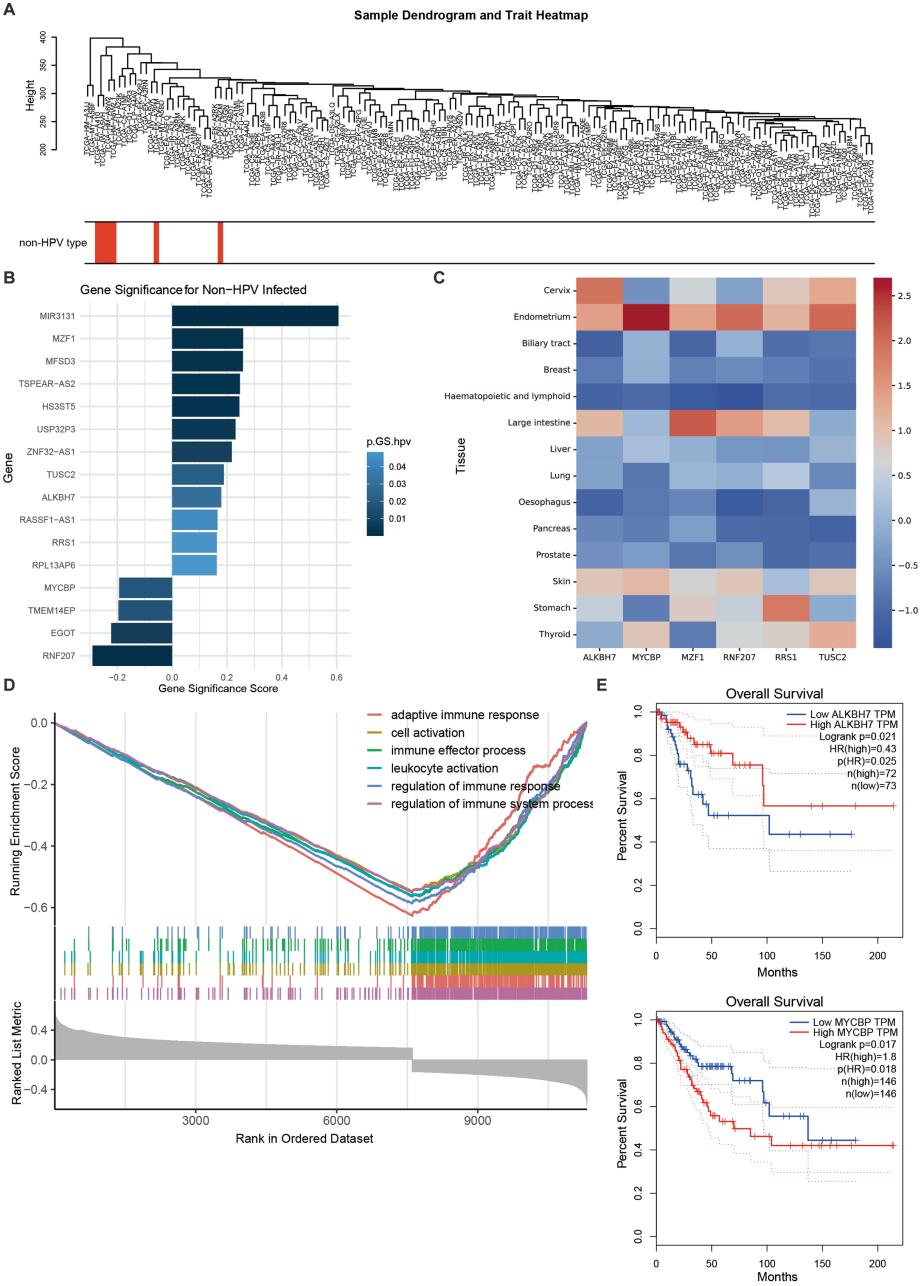
Comprehensive analysis of important genes and their functions in non-HPV-associated cervical cancer. **(A)** Sample dendrogram showing the correlation between sample characteristics and HPV infection; red bars are non-HPV-infected CESCs. **(B)** Nineteen genes that are highly associated with non-HPV infection and mutated in patients. The abscissa indicates the correlation with non-HPV, and a high correlation is a positive value. Colors represent *p*-values. **(C)** Mutation ratios in COSMIC of six genes associated with non-HPV-infected CESC, normalized by z score. **(D)** GSEA enrichment analysis yielded six downregulated pathways with the lowest enrichment scores. **(E)** High expression of ALKBH7 was associated with better prognosis and survival (top), whereas high expression of MYCBP was associated with worse prognosis and survival (bottom).

To identify which types of genes play an important role in non-HPV-infected cervical cancer, we applied weighted gene coexpression network analysis (WGCNA) to analyze genes with common expression patterns in samples. Cluster analysis was performed on all samples, and it was found that the expression patterns of non-HPV-infected samples and HPV-infected samples were different, and the root nodes of non-HPV-infected samples were the same (Figure 4A). The correlation analysis of all genes with HPV infection found that some genes were more correlated with non-HPV infection traits at the transcriptional level, and 19 genes were found to be mutated in patient-1 and patient-3 (Figure 4B; Supplementary Tables S4, S5). Among them, we noticed that six of these genes had high mutation rates in reproductive system cancers in the Catalog of Somatic Mutations in Cancer (COSMIC) database (Figure 4C; Supplementary Table S6).

Genetic changes and the resulting genomic instability have long been recognized as important factors in the mechanisms of cervical cancer progression. The six identified genes with significant differential expression in HPV-negative and HPV-positive patients with CCAC included ALKBH7, MYCBP, MZF1, RNF207, RRS1, and TUSC2. We tried to determine the possible mechanism of how dysregulation of these genes leads to CCAC from previous literature reports. Unfortunately, little research has been done in this area, and we can only speculate from the role of these genes in tumorigenesis in other cancers. Overall, we found that ALKBH7, MYCBP, MZF1, RRS1, and TUSC2 were reported to be involved in the development and progression of cancer.

ALKBH7, a member of the mitochondrial AlkB family, is required for programmed necrosis induced by alkylation and oxidation. Loss of ALKBH7 expression facilitates the restoration of cellular metabolites in response to severe DNA damage, thereby maintaining cell viability (18). ALKBH7 was correlated with the pathological stage of ovarian serous carcinoma and positively correlated with the infiltration of CD8^+^ T cells, dendritic cells, and neutrophils (19). ALKBH7 is also known to be involved in cellular immunity and proliferation of the HeLa cervical cancer cell line (20). Together, ALKBH7 was significantly differentially expressed in different immune and molecular subtypes of multiple tumor types, suggesting that ALKBH7 is a promising pancancer biomarker involved in immune regulation.

Multiple studies have shown that MYCBP plays an oncogenic role in most cancers. In esophageal cancer, miR-26a and miR-26b inhibit the proliferation of tumor cells by inhibiting the expression of MYCBP (21), and the overexpression of MYCBP-binding protein promotes the invasion and metastasis of gastric cancer (22). Qian et al. (23) found that the expression of MYCBP in colorectal cancer tissues was much higher than that in adjacent tissues, and the overexpression of MYCBP promoted the invasion and metastasis of cancer tissues. MZF1 is a transcription factor of the Krupple family of zinc finger proteins (24), and MZF1 has been considered to play an important role in the occurrence, invasion and apoptosis of various tumor cells (25, 26). In lung adenocarcinoma, LKB1-depleted cells promote cancer cell growth, spread, and invasion through the MZF1/MYC axis. Tsai et al. (27) reported that the loss of LKB1 was associated with the upregulation of MYC expression, and this regulatory mechanism may occur at the transcriptional level. As a transcriptional regulator, MZF1 binds to the MYC promoter in LKBH1-deficient cells, upregulates MYC expression, and promotes tumorigenesis.

Abnormal expression of RRS1 negatively affects ribosome synthesis, resulting in abnormal ribosome physiological function. Studies have also shown that the occurrence of tumors is closely related to the abnormal expression of RRS1 (28–30). Gambe et al. (30) used RNA interference technology to downregulate RRS1 expression, and they found that the number of cells in the tetraploid stage increased and the time of cell division was significantly prolonged. This result indicates that the expression level of the RRS1 gene in cervical cancer cells is related to cell proliferation.

Tumor suppressor candidate gene 2 (TUSC2), also known as FUS1, is located on the short arm of human chromosome 3 and is expressed as a tumor suppressor gene in a variety of human cancers (31). Allelic loss and genetic alterations of TUSC2 are present in many cancer types, including lung and breast cancer (32). It has been reported that the upregulation of miR-663 can promote the proliferation, colony formation, migration and invasion of SKOV3 ovarian cancer cells. Meanwhile, the TUSC2 gene was identified as the target gene of miR-663, and its expression was negatively regulated by miR-663, so miR-663 could promote the invasiveness of ovarian cancer cells by targeting TUSC2 (33).

Vanessa’s study found that the expression of RNF207 was upregulated in endometrial cancer, and its expression was higher in lymph node-positive patients than in lymph node-negative patients. Further analysis revealed that RNF207 was directly involved in muscle contraction and ion transmembrane transport (34).

Furthermore, GSEA of all genes more associated with non-HPV-infected traits found that genes inversely associated with non-HPV-infected cervical cancer were also concentrated in immune response and cellular activity (Figure 4D). This suggests that in the absence of HPV invasion, the tumorigenic effect of cervical tumor cells alone is not enough to cause the body’s immune response. In the investigation of these six genes, we found that the relationship between the expression and survival of ALKBH7 and MYCBP was very significant. Among them, highly expressed ALKBH7 was associated with better prognosis and survival, was positively correlated with non-HPV infection, and was also significantly downregulated in cervical cancer. Highly expressed MYCBP was associated with poorer prognosis and survival, was significantly upregulated in cervical cancer, and was more associated with HPV-infected cervical cancer (Figure 4E; Supplementary Figure S1D).

### Characterization and functional enrichment analysis of non-HPV-infected cervical cancer-related modules

Studies have found that HPV-negative cervical cancer can express higher levels of single nucleotide variant (SNV) neoantigens, insertion-deletion (INDEL) neoantigens, and neoantigens associated with cancer/testis antigens (CTA) scores (35). It can be speculated that HPV-negative cervical cancer can be recognized by the immune system through these neoantigens, thereby upregulating immune expression. In addition, high expression of IL-1 was found in tumor tissues of HPV-negative oropharyngeal squamous cell carcinoma (OPSCC). IL-1 acts on NTF IL-1R to stimulate chemokine production, leading to the accumulation of more tumor-associated neutrophils (TANs). In the HPV-negative tumor stroma model, the expression level of the neutrophil-specific chemokine CXCL8 was significantly increased, and this mechanism can also recruit more TANs (35).

**Figure 5:**
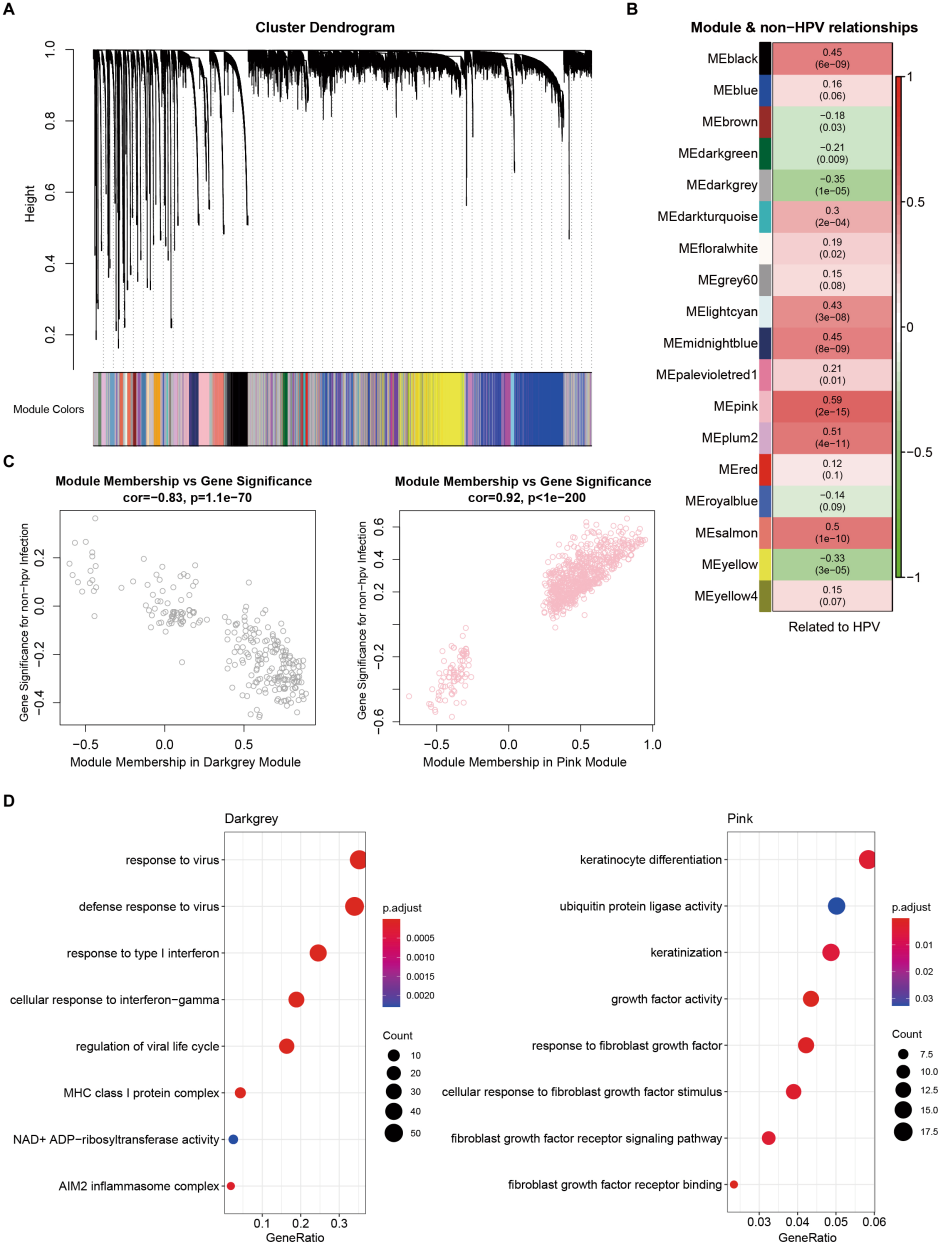
Characterization and functional enrichment of modules associated with non-HPV-infected cervical cancer. **(A)** Network dendrograms from gene coexpression topological overlaps with topological overlap-based differences. Different colors under the tree indicate different modules. Gray indicates genes outside of all modules. **(B)** Associations of module and non-HPV-infected CESC. **(C)** Scatterplot of gene significance (GS) for weights vs. module membership (MM) in dark gray (up) and pink (down) modules. **(D)** GO enrichment analysis of genes from dark gray and pink modules.

To further analyze the related modules, we divided all genes into gene modules with different expression patterns and analyzed non-HPV-infected cervical cancer samples according to each gene set. From the dataset, we identified 30 modules (Figure 5A). We present the modules with the most significant correlations in Figure 5B, and other modules with lesser correlations with HPV infection can be found in Supplementary Table S7. We found that the strongest negative correlation was in the dark gray module, and the strongest positive correlation was in the pink module (Figure 5C). To explore the gene functions contained in the most relevant modules, we performed GO enrichment analysis on the two modules. In the dark gray module, response to virus was the most significant and correlated with the nonviral property of non-HPV-infected cervical cancer. The enrichment of other gene terms related to the killing function of the immune system, including response to type I interferon, cellular response to interferon-gamma, and MHC class I protein complex, showed the characteristics of immune downregulation in cervical cancer without HPV infection, which is mutually consistent with previous conclusions (Figure 5D). In the pink module, the term with the strongest correlation was mainly related to keratinization. Inappropriate keratinization of clear cells was associated with carcinogenesis, and the expression of fibrotic growth factors was also upregulated, which coincided with the fact that most non-HPV-related cervical cancers were clear cell carcinomas, revealing key screening factors in the absence of HPV infection (Figure 5D).

## Discussion

In 1971, Herbst et al. (36) first reported a significant correlation between CCAC and antenatal intrauterine diethylstilbestrol (DES) exposure. The risk of CCAC is approximately 1 in 1,000 among exposed women, most of whom are younger (37). After DES was banned in the 1970s, there has been a significant decrease in DES-related CCAC cases. It is now clear that high-risk HPV is one of the causes of squamous cell carcinoma (SCC) of the cervix, but whether CCAC is related to HPV infection remains controversial. Previous studies have suggested that CCAC may be associated with HPV infection, but many recent studies have noted that CCAC may not be significantly associated with HPV infection (38, 39). Kocken et al. (38) found that among 28 cases of CCAC, HPV was present in 13 cases. However, the expression of HPV was not observed in nine cases of CCAC studied by Park et al. (40). In addition, Ueno et al. (39) also found no correlation between CCAC and high-risk HPV.

In addition to DES exposure, the occurrence of CCAC may also be related to HPV infection, BCL-2 overexpression, and p53 gene mutation (41, 42). Chen et al. (43) reported 21 patients, of which 14 patients had p53 expression, possibly in response to DNA damage, and 18 patients overexpressed BCL-2, and they hypothesized that BCL-2 overexpression could inhibit p53-mediated apoptosis and proposed a mechanism by which these rare tumors can develop without mutations in the p53 gene.

CCAC mostly originates from the Müllerian epithelium and tends to differentiate into the endometrium. Most tumors were less than 4 cm in diameter and were mainly endogenous infiltrative types (44). Gynecological examinations often had no positive findings in early-stage patients, especially for adolescent patients who were not sexually active, and could be easily misdiagnosed or missed because of the inconvenience of vaginal examination. Genetic changes and the resulting genomic instability have long been recognized as important factors in the mechanisms of cervical cancer progression. Herein, we used comparative analysis to screen out genes with large differences in expression between HPV-negative and HPV-positive cervical clear cell carcinoma patients, including ALKBH7, MYCBP, MZF1, RNF207, RRS1, and TUSC2. We tried to find the possible mechanism of CCAC caused by overexpression of these genes from previous literature reports. Unfortunately, studies in this area are very rare, and we can only speculate from the role of these genes in the development of other cancers.

Recent studies have identified the role of ALKBH7 in the progression of several cancers and its relationship to immune cell infiltration. Studies have demonstrated that activation of ALKBH7 in apoptosis-resistant cancer cells may contribute to enhancing the toxic effects of chemotherapeutic alkylating agents in tumors, suggesting a potential alternative pathway for tumor eradication. In addition, there were significant differences in the expression of ALKBH7 among different immune and molecular subtypes of breast cancer (BRCA), prostate adenocarcinoma (PRAD), and uterine corpus endometrial carcinoma (UCEC) (45), suggesting that ALKBH7 may play a role in tumor growth and development. As the target gene of long noncoding RNA (lncRNA), MYCBP can promote the occurrence and development of cancer under the regulation of lncRNA. As a tumor suppressor, Smad4 is inactivated in various cancers (46). As a transcription factor of Smad4, MZF1 directly binds to the core region of the Smad4 promoter, stimulates transcriptional activity, upregulates the expression of Smad4, and then inhibits the migration ability of gastric adenocarcinoma cells (47).

Ribosomal synthesis regulator 1 (RRS1), a 203 amino acid nuclear protein, is essential for ribosome synthesis, and it regulates the rate of ribosome synthesis according to the cellular state, thereby maintaining cellular homeostasis. RRS1 is known to be upregulated in cervical cancer tissue, inhibits apoptosis, and promotes cancer cell proliferation, metastasis and invasion. In addition, it was also found that the expression level of the RRS1 gene in hepatocellular carcinoma tissue was significantly higher than that in adjacent tissue, and the RRS1 gene was related to the proliferation, apoptosis, invasion and metastasis of liver cancer SMMC-7721 cells. Recent studies have also shown that RRS1 is overexpressed in liver and colon cancers and is associated with tumor proliferation.

The TUSC2 gene encodes a multifunctional protein that plays an important role in regulating various cellular processes, such as cell cycle arrest and apoptosis, regulates the functions of various kinases, such as EGFR, PDGFR, AKT, and c-ABL, and affects gene expression. A cellular experiment by Mariniello et al. (48) demonstrated that TUSC2 overexpression inhibited thyroid cancer cell proliferation, migration, and invasion and that TUSC2 repair inhibited tumor cell growth by arresting cell cycle progression and by reducing the motility phenotype of thyroid cancer cell lines. Studies have also found that TUSC2 is downregulated in nasopharyngeal carcinoma and negatively regulates cell proliferation and cell cycle progression (49).

Altogether, ALKBH7, MYCBP, MZF1, RRS1, and TUSC2 were reported to be involved in the development and progression of cancer, and dysregulation of these genes was known to be highly correlated with tumorigenesis. Furthermore, pathway analysis showed that downregulated genes in non-HPV cervical cancer were mainly related to the immune system response, suggesting that non-HPV cervical cancer differs from HPV-infected cervical cancer in that the immune response is weaker, which is consistent with the weak correlation with viral infection. This study may contribute to the screening and further research of HPV-free cervical cancer. Further studies will be needed to explore the pathogenesis of cervical clear cell adenocarcinoma and guide follow-up treatment and prevention.

## Data availability statement

The original contributions presented in the study are included in the article/Supplementary material, further inquiries can be directed to the corresponding authors.

## Ethics statement

The studies involving human participants were reviewed and approved by Department of Gynecology, First Affiliated Hospital of Zhengzhou University, Zhengzhou, China. Written informed consent to participate in this study was provided by the participants’ legal guardian/next of kin. Written informed consent was obtained from the minor(s)’ legal guardian/next of kin for the publication of any potentially identifiable images or data included in this article.

## Author contributions

YS, MZ, and JL: conceptualization. YZ, SC, LZ, and HW: data curation. YS, YZ, MZho, RZ, and JL: writing. MZho, RZ, and DZ: literature review. TZ, XL, CZ, BW, SY, MZha, YZho, and LC: clinical support. YS, MZ, and JL: supervision. All authors contributed to the article and approved the submitted version.

## Funding

This research was supported by grants the National Natural Science Foundation of China (32270611), as well as a grant from Beijing Natural Science Foundation Haidian Origination and Innovation Joint Fund (L222007).

## Conflict of interest

The authors declare that the research was conducted in the absence of any commercial or financial relationships that could be construed as a potential conflict of interest.

## Publisher’s note

All claims expressed in this article are solely those of the authors and do not necessarily represent those of their affiliated organizations, or those of the publisher, the editors and the reviewers. Any product that may be evaluated in this article, or claim that may be made by its manufacturer, is not guaranteed or endorsed by the publisher.

## Footnotes

^1^https://github.com/s-andrews/FastQC
