# Supplementary material for: Genetic alterations in juvenile cervical clear cell adenocarcinoma unrelated to human papillomavirus
Source: Front Med (Lausanne). 2023 Aug 16;10:1211888. doi: 10.3389/fmed.2023.1211888 (PMC10466801; doi:10.3389/fmed.2023.1211888)
Supplement: Supplementary file 1 [file Data_Sheet_1.pdf]

Supplementary Material

Genetic Alterations in Juvenile Cervical Clear Cell Adenocarcinoma Unrelated to Human Papillomavirus

Yuehui Su[1]†, Yiming Zhang[2,3]†, Mengjiao Zhou[1], Ruijin Zhang[2,3], Siang Chen[2,3], Lili Zhang[2], Hao Wang[2,3], Dongdong Zhang[2], Ting Zhang[1], Xinqiang Li[1], Chunyan Zhang[1], Bingjie Wang[1], Shuyu Yuan[1], Mengzhuo Zhang[1], Yingying Zhou[1], Lili Cao[1], Mengzhen Zhang[1*] and Jianjun Luo[2,3,4*]

[1]Department of Gynecology, First Affiliated Hospital of Zhengzhou University, Zhengzhou, China

[2]Key Laboratory of RNA Biology, Institute of Biophysics, Chinese Academy of Sciences, Beijing, China

[3]College of Life Sciences, University of Chinese Academy of Sciences, Beijing, China

[4]Key Laboratory of Epigenetic Regulation and Intervention, Chinese Academy of Sciences, Beijing, China

*** Correspondence:**
Mengzhen Zhang[*], Jianjun Luo[*]
13683806877@163.com (M.Z.), luojianj@ibp.ac.cn (J.L.)

Supplementary Figure and Tables

Supplementary Figure

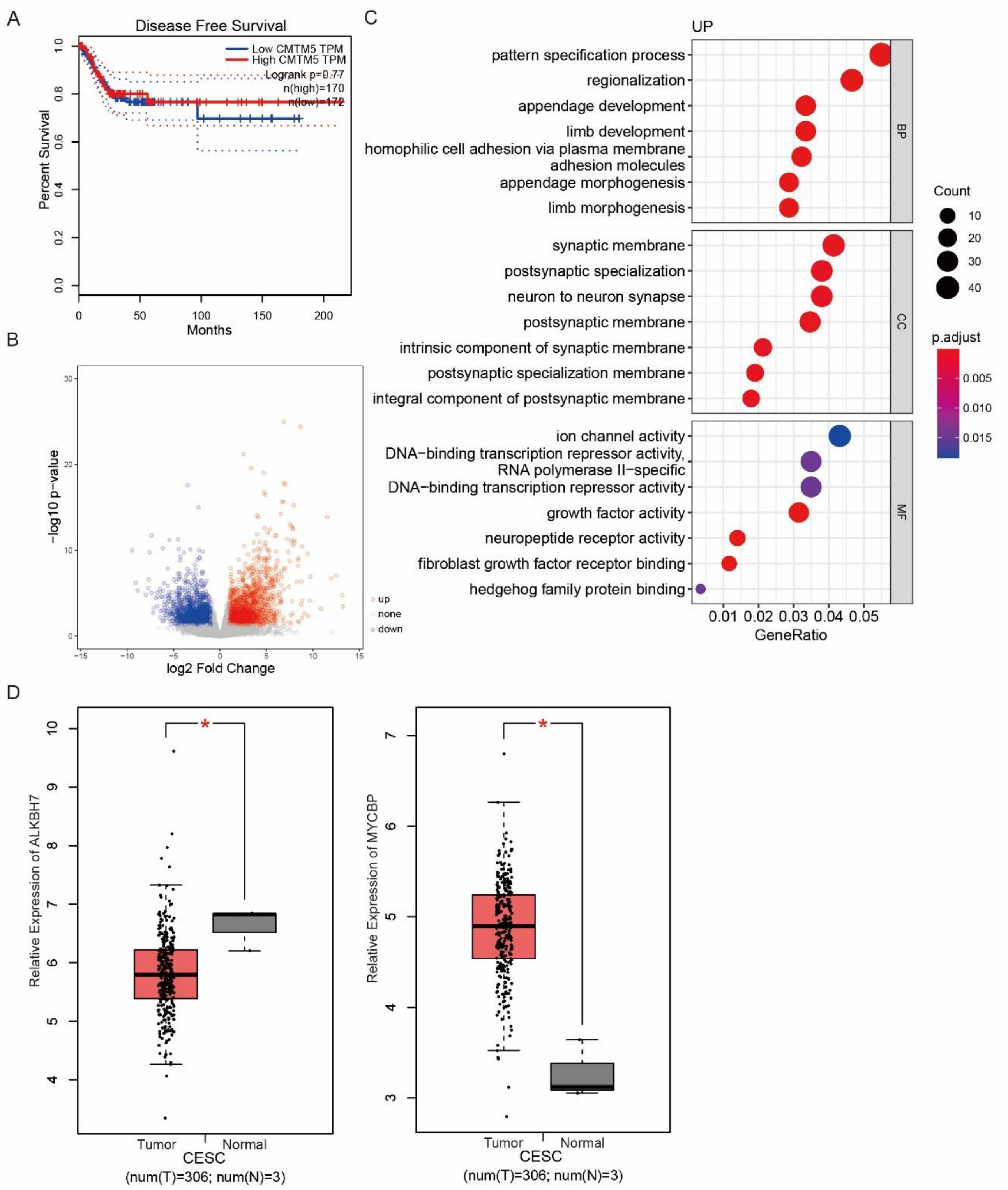

Supplementary Figure S1. Mutated gene CMTM5 and enrichment analysis. (A) Disease free survival (DFS) analysis based on gene expression from GEPIA. Uses Log-rank test, a.k.a the Mantel–Cox test, for hypothesis test. (B) GO enrichment analysis of the most differentially upregulated pathways. (C) Volcano plot of identified differentially expressed genes (DEGs) between HPV infected CESC and

non-HPV infected CESC group. (D) Analysis of data from TCGA and GTEx for comparison of ALKBH7 and MYCBP expression levels in cervical cancer and normal samples, the gene expression levels were shown as log2(TPM+1). *P<0.05.

Supplementary Tables

Supplementary Table S1

Diagnosis and treatment details of three clear cell carcinoma used in this study

Case 1	Age	14
	Initial symptoms	Irregular vaginal bleeding for more than 1 year.
	Diagnosed	MRI: Cervical cancer involving the vagina.<br>Pathology: Cervical clear cell carcinoma.
	Treatment	Hysteroscopy + vaginal tumor resection was performed, and 3 courses of PT were given after surgery: paclitaxel + nedaplatin, followed by radical cervical cancer surgery, and 28 postoperative radiotherapy sessions, including 6 sessions of cisplatin 50mg sensitizing radiotherapy.
	Notes	NA
	Prognosis	Four months after the operation, re-examination of CT considered pulmonary metastasis, and died one year after the operation.
Case 2	Age	6
	Initial symptoms	Vaginal discharge, vaginal bleeding.
	Diagnosed	MRI: cervical solid mass. Pathology: Cervical clear cell carcinoma.
	Treatment	Hysteroscopic resection of cervical tumor was performed, followed by PT regimen: 4 courses of docetaxel + oxaliplatin.
	Notes	After 2 years of recurrence, "laparoscopic hysterectomy + bilateral adnexectomy + pelvic lymph node dissection was performed, and the PT regimen was given 6 times of chemotherapy after surgery: docetaxel + lobaplatin.
	Prognosis	No recurrence was found in PET-CT 4 years after operation.
Case 3	Age	14
	Initial symptoms	Vaginal bleeding.
	Diagnosed	MRI:Upper vagina and cervical space occupying, consider malignant lesions. Pathology: Malignant neoplasm of vaginal growth, prone to cancer, immunohistochemistry suggests the possibility of clear cell carcinoma.
	Treatment	Hysteroscopy + vaginal tumor resection, and postoperative PT program: docetaxel intravenous infusion + lobaplatin for 3 courses of treatment, followed by "laparoscopic uterus + bilateral adnexectomy + pelvic lymph node dissection" , 6 courses of PT regimen were given after the operation.
	Notes	NA
	Prognosis	2 years after operation, PET-CT examination showed no abnormality.

Supplementary Table S2

Genes identified with genetic alterations from patients

Patient-1	Patient-2	Patient-3
ALKBH7	BCL2L12	ASAP1-IT1
APOE	CDK5R1	BAP1
ARFIP2	CT45A6	CENPX
ASAP1-IT2	CT45A7	CMTM5
ATP6AP1	F8A2	CXorf49B
CBR1	F8A3	DDT
CMTM5	KATNBL1P6	GSTT1-AS1
DNAJB7	MIR1184-1	HIST2H2AA3
EGOT	MIR1184-2	LOC554249
EID1	MIR1184-3	MIR567
ENKD1	MIR147B	NTHL1
H2AFZ	MIR6770-1	PDF
HERC2P4	MIR6770-2	SNHG3
HNRNPUL2	MIR6770-3	SNORD116-10
HS3ST5	RPA4	
HTRA2	RPL36AL	
IFRD2	SMCO3	
KDM5D	U2AF1	
KIAA0754	USP17L3	
LINC00216		
LINC01957		
LOC100128361		
LOC100129046		
LOC100131496		
LOC100132356		
LOC105379192		
LOC148696		
LOC401052		
LOC729739		
LRRC24		
LXN		
MFSD3		
MIR1293		
MIR1908		
MIR3131		
MIR3671		
MIR4632		
MIR497		
MIR548AK		

MIR638		
MIR663A		
MIR6724-1		
MIR6724-2		
MIR6724-3		
MIR6726		
MIR8063		
MYCBP		
MZF1		
NAT6		
NSA2		
OR4F16		
OR4F3		
PPP3CB-AS1		
RASSF1-AS1		
RNF207		
RNU105B		
RPL13AP6		
RRS1		
S100G		
SCARNA9L		
SLC39A7		
SNORA24		
SNORA67		
SNORA71D		
SNORA72		
SNORA9B		
SNORD88A		
SPINK13		
STX18-IT1		
SULT1A3		
SULT1A4		
SYP-AS1		
TCEB3C		
TLX1		
TMEM14EP		
TMEM258		
TMEM88		
TOP1P1		
TPRG1-AS2		
TSPEAR-AS2		
TTTY10		
TTTY4		
TUSC2		
U2AF1		

USP32P3		
ZNF32-AS1		

Supplementary Table S3

Mutation point identified within CMTM5 from patients

	Chrom	Pos	Ref	Alt	Symbol
patient-1	chr14	23379102	C	T	CMTM5
patient-3	chr14	23379708	A	T	CMTM5

Supplementary Table S4

Genes related to non-HPV infection, have mutation identified from patient-1

GS.hpv	p.GS.hpv	gene_name
0.1791839	2.93E-02	ALKBH7
-0.222192	6.64E-03	EGOT
0.2452161	2.67E-03	HS3ST5
0.2581975	1.53E-03	MFSD3
0.606546	3.08E-16	MIR3131
-0.1933429	1.86E-02	MYCBP
0.2587439	1.50E-03	MZF1
0.1653889	4.46E-02	RASSF1-AS1
-0.2906243	3.39E-04	RNF207
0.1625284	4.84E-02	RPL13AP6
0.1629806	4.78E-02	RRS1
-0.1956148	1.72E-02	TMEM14EP
0.2473505	2.44E-03	TSPEAR-AS2
0.1887228	2.16E-02	TUSC2
0.2311582	4.70E-03	USP32P3
0.2175325	7.91E-03	ZNF32-AS1

Supplementary Table S5

Genes related to non-HPV infection, have mutation identified from patient-3

GS.hpv	p.GS.hpv	gene_name
0.168485	4.07E-02	PDF
0.2065406	1.18E-02	SNHG3
0.4012452	4.33E-07	SNORD116-10

Supplementary Table S6

Genes harboring point mutation ratio across the primary tissue types that are curated by COSMIC.

	ALKBH7	MYCBP	MZF1	RNF207	RRS1	TUSC2
Cervix	1.20%	0.30%	1.80%	1.20%	0.90%	0.60%
Endometrium	1.02%	1.40%	2.42%	3.05%	1.02%	0.76%
Biliary tract	0.12%	0.46%	0.46%	1.39%	0.12%	0.12%
Breast	0.25%	0.44%	0.76%	0.94%	0.22%	0.11%
Haematopoietic and lymphoid	0.13%	0.12%	0.31%	0.25%	0.12%	0.08%
Large intestine	0.91%	0.49%	3.06%	2.60%	0.98%	0.28%
Liver	0.44%	0.53%	1.27%	1.09%	0.35%	0.31%
Lung	0.44%	0.18%	1.38%	1.34%	0.69%	0.17%
Oesophagus	0.13%	0.19%	0.81%	0.38%	0.06%	0.31%
Pancreas	0.30%	0.20%	1.09%	0.60%	0.10%	0.05%
Prostate	0.35%	0.35%	0.65%	1.01%	0.10%	0.10%
Skin	0.84%	0.84%	1.85%	2.13%	0.62%	0.50%
Stomach	0.70%	0.21%	1.96%	1.82%	1.33%	0.28%
Thyroid	0.48%	0.77%	0.71%	1.92%	0.87%	0.58%

Supplementary Table S7

Associations of module and non-HPV infected CESC

Row.names	correlation	pvalue			
MEantiquewhite2	0.073744	0.373066			
MEantiquewhite4	-0.01546	0.852057			
MEbisque4	0.034738	0.675104			
MEblack	0.454986	6.26E-09			
MEblue	0.155532	0.059081			
MEblue2	-0.00857	0.917671			
MEblue4	0.021027	0.799755			
MEblueviolet	-0.02763	0.738861			
MEbrown	-0.17721	0.031191			
MEbrown2	0.019651	0.81261			
MEbrown4	-0.0282	0.733711			
MEcoral	-0.01846	0.823783			
MEcoral1	-0.01133	0.891263			
MEcoral2	-0.00653	0.937213			
MEcoral3	-0.0068	0.934629			
MEcyan	0.013557	0.870094			
MEdarkgreen	-0.21327	0.009254			
MEdarkgrey	-0.35118	1.21E-05			
MEdarkmagenta	0.056367	0.496213			

MEdarkolivegreen	0.030744	0.710688			
MEdarkolivegreen2	-0.02315	0.780073			
MEdarkolivegreen4	-0.00295	0.971659			
MEdarkorange	0.031586	0.703129			
MEdarkorange2	0.028866	0.727642			
MEdarkred	-0.00352	0.966179			
MEdarkseagreen3	-0.00155	0.985128			
MEdarkseagreen4	-0.01632	0.843883			
MEdarkslateblue	-0.02344	0.777369			
MEdarkturquoise	0.299009	0.000223			
MEdarkviolet	-0.00998	0.904154			
MEdeeppink	-0.03043	0.713545			
MEfirebrick3	-0.02001	0.809231			
MEfirebrick4	-0.02594	0.754325			
MEfloralwhite	0.185723	0.023824			
MEgreen	0.005695	0.94523			
MEgreenyellow	0.001569	0.984904			
MEgrey	0.056334	0.496462			
MEgrey60	0.146636	0.075338			
MEhoneydew	0.012691	0.878332			
MEhoneydew1	0.020799	0.80188			
MEindianred3	0.014246	0.863555			
MEindianred4	-0.00814	0.921759			
MEivory	-0.0029	0.972117			
MElavenderblush1	0.010647	0.897805			
MElavenderblush2	0.017002	0.837491			
MElavenderblush3	0.029103	0.725495			
MElightblue4	-0.02079	0.801977			
MElightcoral	-0.00065	0.993734			
MElightcyan	0.434975	3.31E-08			
MElightcyan1	-0.01841	0.824274			
MElightgreen	-0.01644	0.842788			
MElightpink2	0.005924	0.943036			
MElightpink3	-0.01462	0.859996			
MElightpink4	-0.00317	0.969505			
MElightslateblue	0.008553	0.917826			
MElightsteelblue	-0.04241	0.60877			
MElightsteelblue1	-0.01738	0.833934			
MElightyellow	-0.00334	0.967855			
